# Antibiotic resistant *Escherichia coli* from diarrheic piglets from pig farms in Thailand that harbor colistin-resistant *mcr* genes

**DOI:** 10.1038/s41598-022-13192-3

**Published:** 2022-05-31

**Authors:** Luong Thi Yen Nguyet, Krittika Keeratikunakorn, Kampon Kaeoket, Natharin Ngamwongsatit

**Affiliations:** 1 Department of Clinical Sciences and Public Health, Faculty of Veterinary Science, Mahidol University, Nakhon Pathom, Thailand; 2 Laboratory of Bacteria, Veterinary Diagnostic Center, Faculty of Veterinary Science, Mahidol University, Nakhon Pathom, Thailand

**Keywords:** Microbiology, Molecular biology

## Abstract

Antibiotic-resistant *Escherichia coli* is one of the most serious problems in pig production. This study aimed to determine the antibiotic susceptibility and genotypes profiles of diarrhoeagenic *E. coli* that causes diarrhea in piglets. Thirty-seven pathogenic *E. coli* strains were used in this study. These were isolated from rectal swabs of diarrheic piglets from farms in Thailand from 2018 to 2019. *Escherichia coli* isolates were highly resistant to amoxicillin (100%), followed by oxytetracycline (91.9%), enrofloxacin (89.2%), trimethoprim/sulfamethoxazole (86.5%), amoxicillin: clavulanic acid (81.1%), colistin and gentamicin (75.7%), ceftriaxone and ceftiofur (64.9%), ceftazidime (35.1%) and 97.3% showed multidrug-resistance (MDR). There were 8 (21.6%) *mcr-1* carriers, 10 (27.0%) *mcr-3* carriers and 10 (27.0%) co-occurrent *mcr-1* and *mcr-3* isolates. The phenotype-genotype correlation of colistin resistance was statistically significant (performed using Cohen’s kappa coefficient (κ = 0.853; *p* < 0.001)). In addition, PCR results determined that 28 of 37 (75.7%) isolates carried the *int1* gene, and 85.7% *int1*-positive isolates also carried the *mcr* gene. Genetic profiling of *E. coli* isolates performed by ERIC-PCR showed diverse genetics, differentiated into thirteen groups with 65% similarity. Knowledge of the molecular origins of multidrug-resistant *E. coli* should be helpful for when attempting to utilize antibiotics in the pig industry. In terms of public health awareness, the possibility of transmitting antibiotic-resistant *E. coli* from diarrheic piglets to other bacteria in pigs and humans should be of concern.

## Introduction

*E. coli* is one of the main causes of diarrheal disease in neonatal and weaned piglets. This disease is a financial burden in the pig production system due to high mortality rates, decreased weight gain, high cost for treatments, vaccinations, and feed supplements, and it accounts for 11.5–29.5% of piglet deaths worldwide^1, 2^. According to its pathogenic potential, *E. coli* is separated into non-pathogenic and pathogenic groups. The non-pathogenic *E. coli* is a commensal present in the intestinal lumen^3^. Pathogenic *E. coli* are causative agents of intestinal and extraintestinal diseases in humans and animals. *Escherichia coli* strains are classified into different “pathotypes” based on the presence of virulence factors. There are six types of diarrhoeagenic *E. coli*, including enterotoxigenic *E. coli* (ETEC), enteropathogenic *E. coli* (EPEC), Shiga toxin-producing *E. coli* (STEC), enteroinvasive *E. coli* (EIEC), enteroaggregative *E. coli* (EAEC), and diffusely adherent *E. coli* (DAEC) that causes diarrhea in young pigs^4, 5^. An outbreak of *E. coli* frequently requires quick action, and therefore antibiotics are used in the pig industry to control diarrhea caused by *E. coli*.

Unfortunately, using antibiotics for decades led to developed antimicrobial resistance in pathogens and caused risks of transmitting antimicrobial resistance genes transmission in the environment. Presently, antimicrobial resistance (AMR) is rising to dangerously high levels, while it is causing public health issues worldwide. For example, antibiotics are becoming less effective in the treatment of common infectious diseases in humans^6^. The type of antibiotics frequently used in diarrhea caused by *E. coli* in pigs are β-lactam antibiotics (amoxicillin and a combination containing amoxicillin/clavulanic acid), cephalosporins (ceftiofur, cefquinome), aminoglycosides (apramycin, neomycin, gentamicin), aminocyclitols (spectinomycin) sulfonamide combined with trimethoprim (such as trimethoprim/sulfamethoxazole), fluoroquinolones (enrofloxacin, marbofloxacin and danofloxacin), quinolones (flumequine) and polymyxins (colistin sulfate)^7^.

Colistin (also known as polymyxin E) is an antibiotic commonly used against Gram-negative bacteria in pig production to prevent and treat diarrhea caused by *E. coli*^8^*.* Excessive utilization of colistin in animal production has created selective pressure contributing to the increased resistance to colistin. In fact colistin resistance brought about colistin-resistant genes in the *mcr* family^9^. Since the first *mcr-1* gene was reported in 2016, so far ten *mcr* variants have been described^10^. Among them, *E. coli* was reported to harbor the *mcr-1*, *mcr-2*, *mcr-3*, *mcr-4* and *mcr-5* genes^11^. The *mcr-1* gene is the most frequently detected in different animal food species and from *Enterobacteriaceae* infections in humans^9^. Strains carrying antibiotic-resistance genes can transmit resistance genes to other pathogens. Horizontal gene transfer is a major mechanism for increased antimicrobial resistance, but it is more rapid than simple mutations. Resistance genes are often associated with mobile genetic elements such as plasmids, transposons, and integrons that facilitate the integration and spread of resistance genes^12^. The class 1 integron-integrase gene (*int1*) plays a major role in the dissemination of antibiotic resistance. It is a genetic mechanism that allows bacteria to acquire, store and express new genes^13^.

Resistance of *E. coli* isolates from pig farms to a wide range of antimicrobial agents has dramatically increased over several years worldwide^14^. Studies from pig production farms by García et al.^8^ showed 91.6% resistance to at least three different antimicrobial classes, van Breda et al.^15^ showed that over 70% of isolates were resistant to antibiotics commonly used in veterinary practice, Jiang et al.^16^ showed 86.2% multidrug-resistance (MDR), and Khine et al.^9^ showed all 31 *mcr-*positive *E. coli* (MCRPE) were MDR. Therefore, the objective of this study was to investigate the antibiotic resistance of *E. coli* isolated from diarrheic piglets in Thailand and to identify the presence of *mcr* and *int1* genes in all isolates.

## Materials and methods

### *Escherichia coli* collection and virulence genes detection

Thirty-seven pathogenic *E. coli* isolates were previously isolated and identified in routine microbiology service at the Laboratory of Bacteria, Veterinary Diagnostic Center, Faculty of Veterinary Science, Mahidol University. The isolates were obtained from rectal swabs of diarrheic piglets from farms in Thailand during Edema disease outbreak from 2018 to 2019. All isolates were stored at − 80 °C at the Veterinary Diagnostic Center, Faculty of Veterinary Science, Mahidol University, Thailand. Nineteen specific virulence genes of pathogenic *E. coli*, including 7 toxins genes (*lt*, *sth*, *stp*, *stx1A*, *stx2A*, *stx2e* and *astA*), 7 adhesin genes (*bfpA*, *eaeA*, *ipaH*, *aggR*, *pCDV432*, *paa* and *aidA*) and 5 fimbriae genes (*F4*, *F5*, *F6*, *F18* and *F41*) were detected using multiplex PCR. Two groups of primer sets were used to detect and discriminate virulence genes in *E. coli* isolates as listed in Supplementary Table S1. Multiplex PCR was carried out using a BiometraTOne96G thermocycler (AnalytikJena, Germany). For the group A primer (1A and 2A), amplification was performed with an initial denaturation step at 95 °C for 5 min, followed by 35 cycles of denaturation at 95 °C for 1 min, annealing at 52 °C for 1 min, extension at 72 °C for 1 min, and a final extension at 72 °C for 5 min. In the case of amplification with the group B primer (1B, 2B and 3B), PCR was conducted with an initial denaturation step at 95 °C for 5 min, followed by 30 cycles of denaturation at 95 °C for 30 s, annealing at 61 °C, 57 °C, and 56 °C for fimbriae, toxins, and alternate adhesins, respectively, for 45 s, extension at 72 °C for 1 min, and a final extension step at 72 °C for 7 min. The PCR products were separated using 1.5% agarose gel electrophoresis, stained with 1× GelRed (Sigma Aldrich, USA), and visualized under a UV transilluminator UVP GelStudio (AnalytikJena, USA).

### Antimicrobial susceptibility testing

All isolates were tested for antimicrobial susceptibility by broth microdilution according to the guidelines of the Clinical and Laboratory Standards Institute (CLSI) (VET01S)^17^. Broth microdilution in 96-well microdilution plates was used to determine minimal inhibitory concentrations (MICs). The antibiotic stock solution (256 µg/mL) was diluted by serial two-fold dilutions in Mueller Hinton broth (MHB) and a quality control was composed of media without antibiotic. The following antibiotics were tested: amoxicillin (AMX), amoxicillin: clavulanic acid (AMC), ceftiofur (CEF), ceftazidime (CAZ), ceftriaxone (CRO), colistin (CT), enrofloxacin (ENR), gentamicin (CN), oxytetracycline (OTC), trimethoprim/sulfamethoxazole (SXT). The inoculum was prepared by taking colonies from nutrient agar (NA) by a sterile swab and preparing a McFarland standard. The inoculum was dispensed into the microdilution plate with the serialy diluted antibiotic and incubated at 37 °C for 16–20 h. *Escherichia coli* ATCC 25922 was used in each assay as a quality control. Results were recorded as the lowest concentration of an antimicrobial that inhibited visible growth of a microorganism.

The resistance breakpoints of the *E. coli* isolates were as follows: amoxicillin (AMX) (≥ 32 μg/mL), amoxicillin: clavulanic acid (AMC) (≥ 32 μg/mL), ceftiofur (CEF) (≥ 8 μg/mL), ceftazidime (CAZ) (≥ 16 μg/mL), ceftriaxone (CRO) (≥ 4 μg/mL), colistin (CT) (≥ 4 μg/mL), enrofloxacin (ENR) (≥ 2 μg/mL), gentamicin (CN) (≥ 16 μg/mL), oxytetracycline (OTC) (≥ 16 μg/mL) and trimethoprim/sulfamethoxazole (SXT) (≥ 8 μg/mL) according to CLSI guidelines (VET01S, M100)^17, 18^. Multidrug resistance (MDR) was defined “as non-susceptibility to at least one agent in three or more antimicrobial categories”^19^.

### Detection of *mcr *and *int1* genes using multiplex polymerase chain reaction (mPCR)

Plasmid-mediated colistin-resistant genes *mcr-1*, *mcr-2, mcr-3, mcr-4, mcr-5, mcr-6, mcr-7*, *mcr-8, mcr-9* and *mcr-10* were detected by multiplex PCR and the class 1 integron-integrase gene (*int1*) was detected by simplex PCR. mPCR was performed in 2 reactions including reaction 1 (for detecting *mcr-1*, *mcr-4, mcr-5, mcr-7*, *mcr-8* and *mcr-10*) and reaction 2 (for detecting *mcr-2, mcr-3, mcr-5, mcr-6* and *mcr-9*), and all reactions were performed with a positive control (contained genes of *mcr-1* to *10*). Plasmid DNA was extracted from 1 mL of overnight culture using QIAprep Spin Miniprep Kit (Qiagen, Germany) following the manufacturer’s instructions. PCR reactions were performed in a total volume of 20 µL containing 0.4 µM of each forward and reverse primer, 20 ng of DNA template and 1× Green PCR master mix kit (Biotechrabbit, Germany). The primers and the expected size of DNA products are listed in Supplementary Table S1. Amplification steps were accomplished using a BiometraTOne96G thermocycler (AnalytikJena, Germany) with the following thermal cycles: the initial denaturation at 94 °C for 3 min, followed by 25 cycles (denaturation at 94 °C for 30 s, annealing at 58 °C for 90 s, and extension at 72 °C for 60 s), and a final extension step at 72 °C for 5 min. The PCR products were separated using 1.5% agarose gel electrophoresis, stained with 1× GelRed (Sigma Aldrich, USA) and then visualized under a UV transilluminator UVP GelStudio (AnalytikJena, USA).

### Enterobacterial repetitive intergenic consensus polymerase chain reaction (ERIC-PCR) for* E. coli* isolates

ERIC-PCR was performed on a total of 37 *E. coli* isolates with primers ERIC-1 (5′-ATG TAA GCT CCT GGG GAT TCA C-3′) and ERIC-2 (5′-AAG TAA GTG ACT GGG GTG AGC G-3′) that were described in previous studies^20^. Genomic DNA was extracted from 1 mL of overnight culture using a G-spin™ Genomic DNA Extraction Kit (iNtRON, Korea) and following the manufacturer’s instructions. ERIC-PCR was performed in a total volume of 20 μL containing 0.4 μM concentrations of each forward and reverse primer, 20 ng of DNA template and 1× Green PCR master mix kit (Biotechrabbit, Germany). The amplification steps were completed using a BiometraTOne96G thermocycler (AnalytikJena, Germany) with the following thermal cycles: the initial denaturation at 94 °C for 5 min, followed by 35 cycles (denaturation at 94 °C for 1 min, annealing at 52 °C for 1 min, and extension at 72 °C for 5 min), and a final extension step at 72 °C for 10 min. PCR products were separated using 2.0% agarose gel electrophoresis, stained with 1X GelRed (Sigma Aldrich, USA), and visualized under a UV transilluminator UVP GelStudio (AnalytikJena, USA). ERIC-PCR results were analyzed by online data analysis services (insilico.ehu.es). ERIC profiles were compared using the Dice coefficient method, and a dendrogram was made via the unweighted pair-group method using arithmetic averages (UPGMA).

### Statistical analysis

Genotype–phenotype correlations of harboring *mcr* genes and colistin resistance in *E. coli* isolates were performed using Cohen’s kappa coefficient in SPSS version 23 (IBM Corp. in Armonk, NY). *p* value of < 0.05 were considered statistically significant.

### Ethics approval and consent to participate

The study was carried out in compliance with the ARRIVE guidelines. This research project was approved by the Faculty of Veterinary Science-Animal Care and Use Committee (FVS-ACUC-Protocol No. MUVS-2019-06-31 and MUVS-2021-10-40). All methods were performed in accordance with the relevant guidelines and regulations.

## Results

### Detection of virulence genes in *E. coli*

The distribution of virulence genes in each *E. coli* isolate are presented in Table 1. Among the 37 *E. coli* isolates examined, 30 (81.1%) were found to harbor virulence genes. Ten genes encoding *astA*, *lt*, *stp*, *sth*, *stx2e*, *aidA*, *eaeA*, *F4*, *F18* and *F41* were detected. The prevalence of toxins genes, *astA*, *lt*, *stp*, *sth*, *stx2e*, was 51.3, 24.3, 18.9, 5.4 and 2.7%, respectively. The prevalence of the adhesin genes, *eaeA* and *aidA*, was 18.9 and 5.4%, respectively. The prevalence of fimbriae genes, *F18*, *F4* and *F41*, was 16.2, 2.7 and 2.7%, respectively. In contrast, the genes encoding *stx1A*, *stx2A*, *ipaH*, *bfpA*, *pCVD432*, *aggR*, *paa*, *F5* and *F6* were not detected in any of the 37 isolates examined.

**Table 1:**
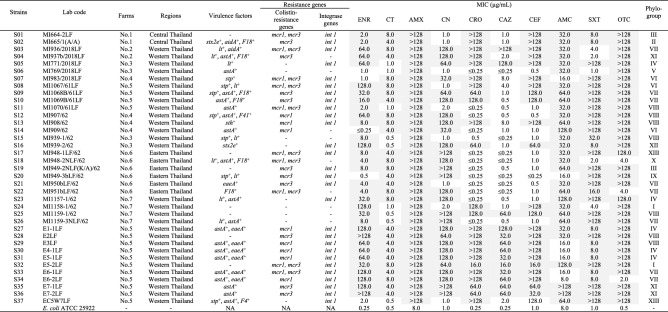
Characteristics and antimicrobial susceptibility against 10 antimicrobial agents of *E. coli* isolated from the rectal swabs of diarrheic piglets of farms in Thailand during 2018 to 2019.

Virulence factors included in this data table: shiga-toxin (*stx2e*), heat-labile enterotoxin (*lt*), heat-stable enterotoxin (*stp, sth*), enteroaggregative *E. Coli* heat-stable enterotoxin 1 (*astA*), intimin (*eaeA*), adhesin (*aidA*)*,* fimbriae (*F4*, *F18*, *F41*).

The values were below or above the dilution range marked with the sign “ ≤ ” or “ > ”.

MICs highlighted in light grey represent resistance according to CLSI (VET01S, M100) guidelines.

NA means not analyzed.

*AMX* amoxicillin, *AMC* amoxicillin: clavulanic acid, *CEF* ceftiofur, *CAZ* ceftazidime, *CRO* ceftriaxone, *CT* colistin, *ENR* enrofloxacin, *CN* gentamicin, *OTC* oxytetracycline, *SXT* trimethoprim/sulfamethoxazole.

### Antimicrobial susceptibility testing

**Figure 1:**
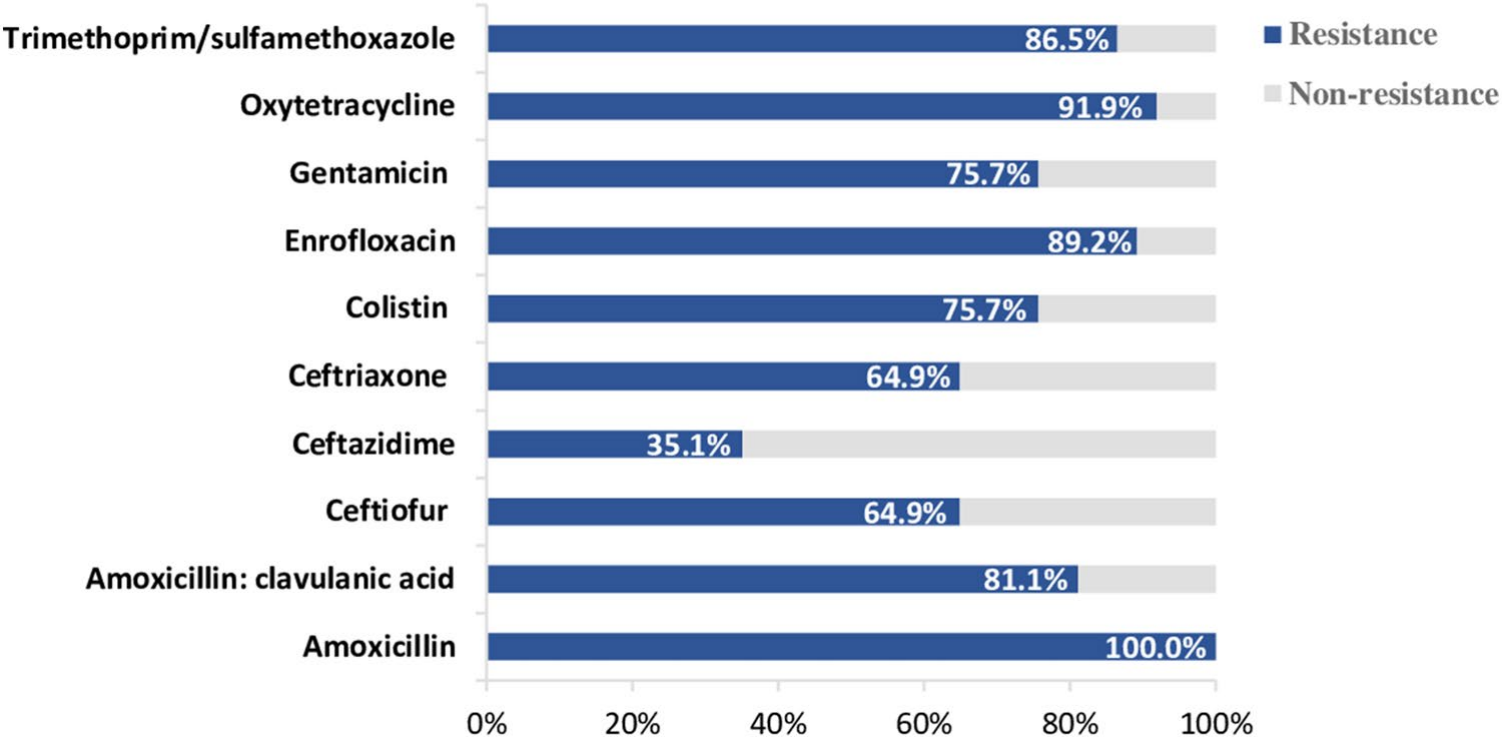
The antimicrobial-resistance percentages of *E. coli* strains (N = 37). Antimicrobial susceptibility was performed by MIC assay and analyzed based on the resistance breakpoints according to CLSI (VET01S, M100) guidelines.

Thirty-seven pathogenic *E. coli* isolates were tested for antimicrobial susceptibility to 10 different antibiotics (Table 1). All of the samples were resistant towards amoxicillin (100%) followed by oxytetracycline (91.9%), enrofloxacin (89.2%), trimethoprim/sulfamethoxazole (86.5%), amoxicillin:clavulanic acid (81.1%), colistin and gentamicin (75.7%), ceftriaxone and ceftiofur (64.9%) and ceftazidime (35.1%) (Fig. 1). The results showed that 36 of 37 isolates (97.3%) were resistant to at least four different antimicrobial classes, which indicates multidrug-resistance (MDR). About 75.68% showed resistance to β-lactams, fluoroquinolone and aminoglycosides/polymyxin E, and 45.95% of isolates were resistant to all seven antimicrobial classes with different patterns (Table 2).

**Table 2:** Antibiograms based on the MIC results of 37 *E. coli* strains distributing into 21 pattern types (A-U).

Pattern	Profile	Number of resistance antimicrobials	Isolate(s)
A	AMX-AMC-ENR-OTC-SXT-CT-CN-CRO-CEF-CAZ	10	5
B	AMX-AMC-ENR-OTC-SXT-CN-CRO-CEF-CAZ	9	2
C	AMX-AMC-ENR-OTC-CT-CN-CRO-CEF-CAZ	9	1
D	AMX-AMC-ENR-OTC-SXT-CT-CN-CRO-CEF	9	3
E	AMX-ENR-OTC-SXT-CT-CN-CRO-CEF-CAZ	9	4
F	AMX-AMC-ENR-OTC-SXT-CN-CRO-CEF	8	1
G	AMX-AMC-ENR-OTC-SXT-CT-CRO-CEF	8	3
F	AMX-AMC-ENR-OTC -CT-CN-CRO-CEF	8	1
I	AMX-ENR-SXT-CT-CN-CRO-CEF-CAZ	8	1
J	AMX-AMC-ENR-OTC-SXT-CRO-CEF	7	1
K	AMX-AMC-ENR-OTC-SXT-CT-CN	7	4
L	AMX-OTC-SXT-CT-CN-CRO-CEF	7	1
M	AMX-AMC-ENR-OTC-CRO-CEF	6	1
N	AMX-AMC-ENR-OTC-SXT-CN	6	1
O	AMX-AMC-ENR-OTC-SXT-CT	6	1
P	AMX-AMC-ENR-SXT-CT-CN	6	1
Q	AMX-AMC-OTC-SXT-CT-CN	6	1
R	AMX-AMC-ENR-OTC-SXT	5	2
S	AMX-AMC-ENR-CT-CN	5	1
T	AMX-OTC-SXT-CT-CN	5	1
U	AMX-AMC-OTC	3	1

*AMX* amoxicillin, *AMC* amoxicillin: clavulanic acid, *CEF* ceftiofur, *CAZ* ceftazidime, *CRO* ceftriaxone, *CT* colistin, *ENR* enrofloxacin, *CN* gentamicin, *OTC* oxytetracycline, *SXT* trimethoprim/sulfamethoxazole.

**Figure 2:**
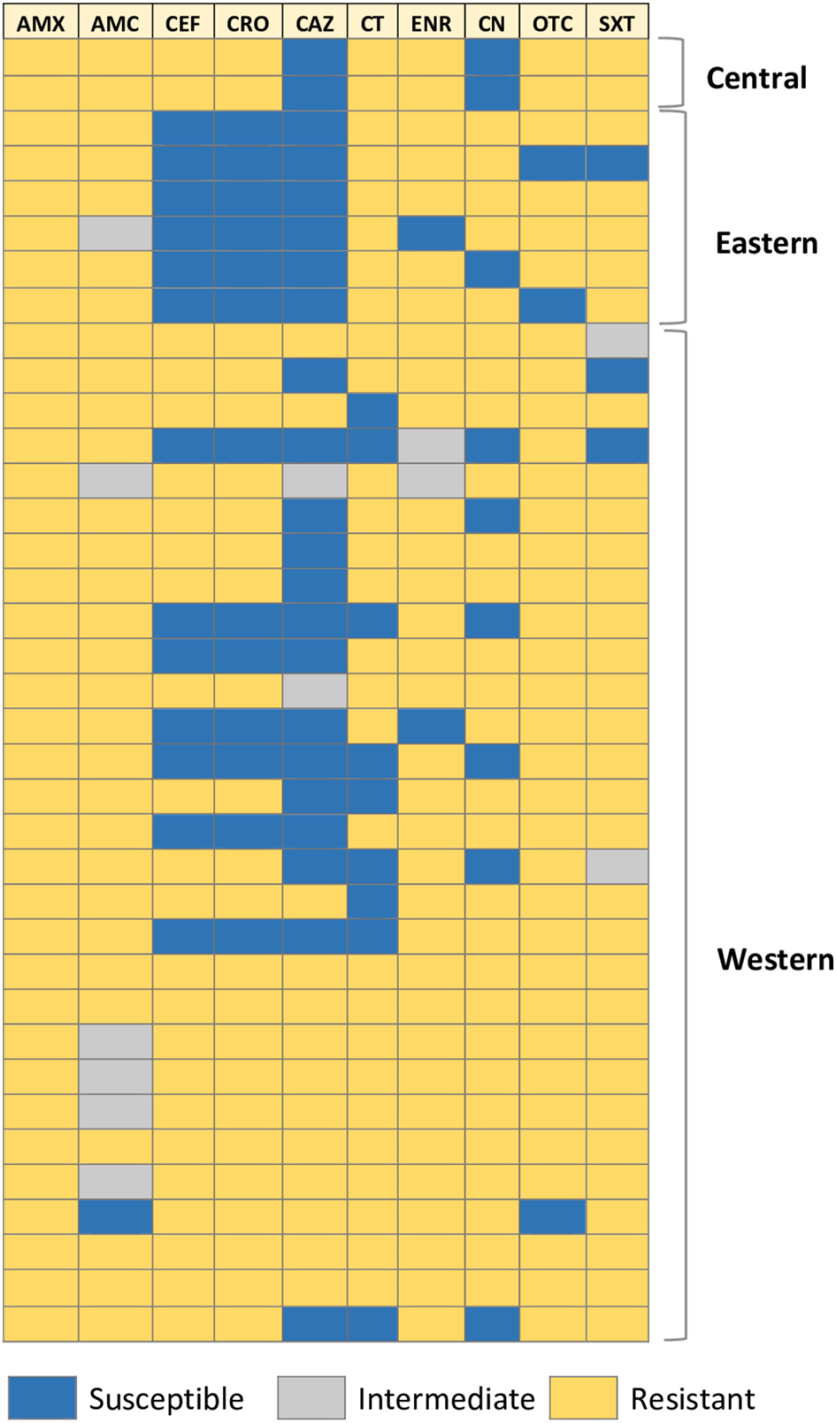
The MIC profiles of 37 *E. coli* isolates from diarrheic piglets. The right vertical axis lists the geographical regions of Thailand and the horizontal axis is labeled with the antibiotics. The color bar represents the antimicrobial susceptibility results.

The majority of *E. coli* isolates (29 of 37) were from Western Thailand, which has the highest pig farm density in Thailand. Minority isolates were from Central and Eastern Thailand (i.e., 2 and 6 isolates, respectively). The result of antimicrobial susceptibility showed resistance to amoxicillin, amoxicillin: clavulanic acid, colistin, enrofloxacin, oxytetracycline, trimethoprim/sulfamethoxazole, which is common across all three regions from 66.7 to 100% resistance (Fig. 2). Isolated *E. coli* (2/2) from Central Thailand were resistant to ceftiofur, ceftriaxone and susceptible to gentamicin, ceftazidime. All of the samples from Eastern Thailand were resistant to gentamicin with 83.3% and 100% of samples (6/6) susceptible towards third-generation cephalosporins including ceftiofur, ceftazidime, and ceftriaxone. *Escherichia coli* isolates from Western Thailand were resistant towards gentamicin (79.3%), ceftiofur (75.9%), ceftriaxone (75.9%) and ceftazidime (44.8%).

### Colistin-resistance genes (*mcr) *and the class 1 integron-integrase gene (*int1*)

A total of 37 pathogenic *E. coli* isolates were determined for colistin-resistance genes *mcr-1* to *10* by multiplex PCR. A total of 28 (75.7%) isolates carried *mcr-1* and *mcr-3* genes (Table 3). None of the isolates carried *mcr-2*, *mcr-4*, *mcr-5*, *mcr-6*, *mcr-7*, *mcr-8*, *mcr- 9* and *mcr-10* genes. *mcr-1* gene was found in 18 of 37 (48.6%) isolates and the *mcr-3* gene was found in 20 of 37 isolates (54.1%). Among 28 *mcr*-positive isolates, 10 (27.0%) isolates carried both *mcr-1* and *mcr-3* genes. The correlation between genotypes and phenotypes of colistin resistance are seen in Table 4. PCR results also determined that 28 of 37 (75.7%) isolates carried the *int1* gene (Table 1). Almost all *int1*-positive isolates (24 isolates) carried the *mcr* gene and only four *int1*-positive isolates did not contain the *mcr* gene.

**Table 3:** The distribution of *E. coli* strains (N = 28/37) harboring colistin-resistance genes at different MICs of colistin.

*mcr* genes	No. of isolates	Different MIC of colistin (%)			
		1 µg/mL	2 µg/mL	4 µg/mL	8 µg/mL
*mcr-1*	8	–	–	5 (17.9%)	3 (10.7%)
*mcr-3*	10	–	–	8 (28.6%)	2 (7.1%)
*mcr-1* + *mcr-3*	10	1 (3.6%)	–	2 (7.1%)	7 (25.0%)
Total	28 (100%)	1 (3.6%)	0	15 (53.6%)	12 (42.9%)

**Table 4:** Comparison between genotypes and phenotypes of colistin resistance using mPCR and MIC assays from 37 *E. coli* isolates, which are based on the resistance breakpoints according to CLSI (VET01S, M100) guidelines.

Colistin	Resistance genotype	Non-resistance genotype	Total
Resistance phenotype	27	1	28
Non-resistance phenotype	1	8	9
Total	28	9	37

### Genetic profiling of *E. coli* isolates

**Figure 3:**
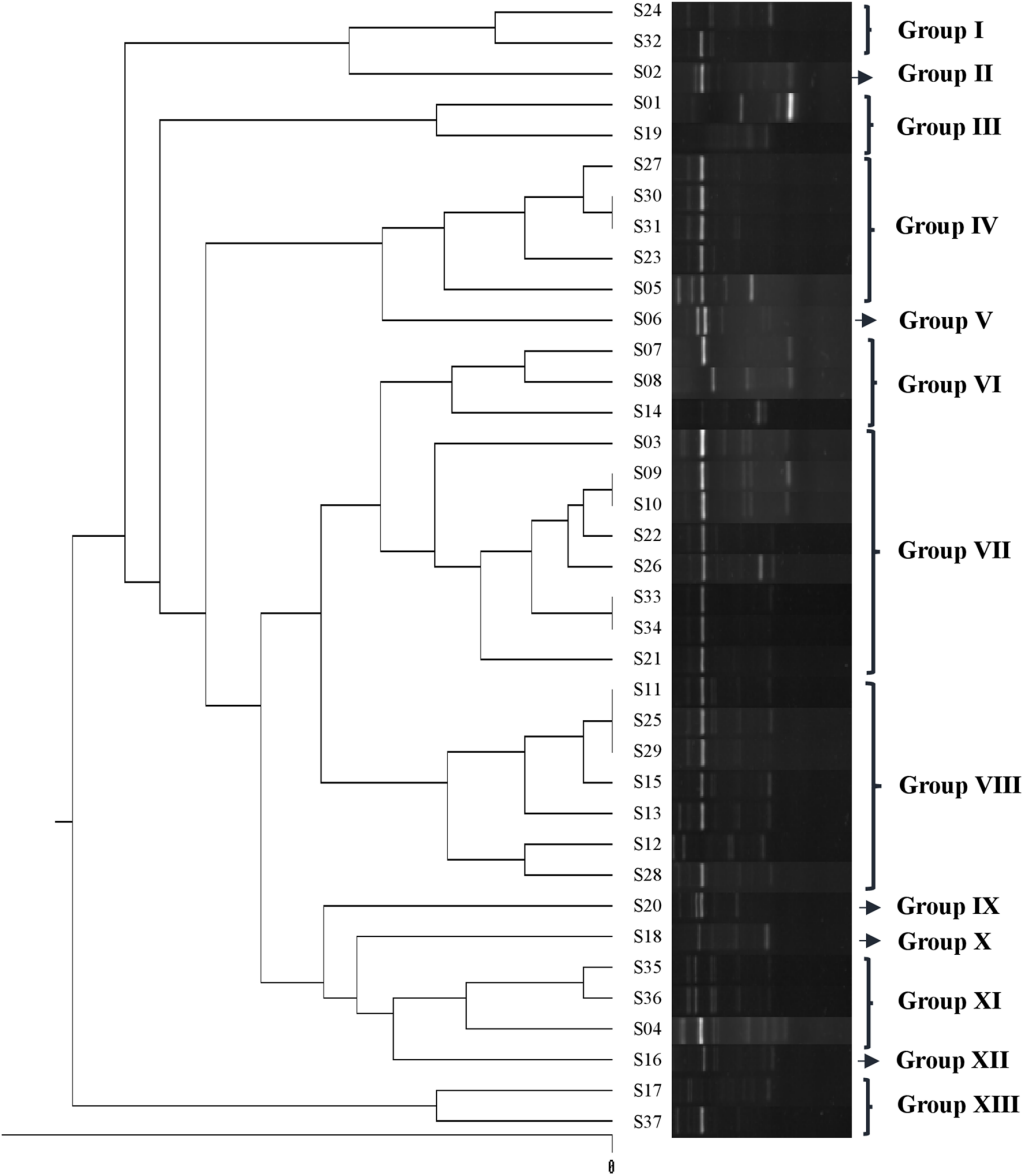
Dendrogram of 37 *E. coli* isolates from ERIC-PCR banding pattern. Similarity analysis was performed by Dice coefficient and UPGMA method. The original ERIC-PCR gels are presented in Supplementary Fig. S1.

ERIC sequences of 37 isolates were amplified using PCR with ERIC-1 and ERIC-2 primers. Bands for each sample were recorded according to their molecular weights based on a molecular marker (100 bp DNA Ladder). All 37 pathogenic *E. coli* isolates had bands and were genotyped. The dendrogram from ERIC-PCR banding pattern was analyzed and the isolates were differentiated into thirteen groups with 65% similarity (Fig. 3). ERIC-PCR profile of some isolates showed a difference from others resulting in separate groups with only one isolate in each group (II, V, IX, X and XII). However, groups IV, VII and VIII had a greater number of isolates with 5, 8 and 7 isolates, respectively.

## Discussion

Diarrheal disease caused by *E. coli* is one of the most common diseases in neonatal and weaned piglets. Although there are many approaches to prevent pathogenic *E. coli* infection in piglets, antibiotics are still commonly used to treat enteric colibacillosis in swine^16^. However, using antibiotics to control infection led to increased selection pressure and resulted in the selection of antibiotic-resistant bacteria. Governments sector worldwide, including Thailand, issued regulations on the use of antibiotics in livestock; however, the risk of antimicrobial resistance gene transmission is still a great global concern. *Escherichia coli* that carry the antimicrobial resistance gene may transfer these to other pathogens and human pathogens, particularly the *mcr* gene which mediates colistin resistance in animals and humans^9^. Genotype and antimicrobial susceptibility-profiles of *E. coli* would be helpful for the clinical use of antibiotics. In this study, we investigated whether or not *E. coli* found in rectal swabs of diarrheic piglets from farms in Thailand were resistant to antimicrobes and if they harbored colistin-resistance *mcr* genes. Our data showed that most of these strains contain one or more virulence factor genes (Table 1).

The result of antimicrobial susceptibility testing showed that 37 *E. coli* isolates were resistant to at least three antibiotics with 21 different patterns. The resistance rate to ceftazidime were the lowest with 35.1% and the resistance rate to amoxicillin were the highest with 100%. 97.3% of the isolates were multidrug-resistant (MDR), which means resistant to at least one agent in three or more antimicrobial categories. Among them, the resistance to β-lactams and fluoroquinolone antimicrobial were the most frequent. Consequently, a diarrheal disease caused by multidrug-resistant *E. coli* (MDR-*E. coli*) can be difficult to treat in pig farms. These results contribute to the overall picture of antimicrobial resistance in *E. coli* in the pig industry. Studies from many countries showed that MDR-*E. coli* isolates from pigs were 86.2% in US (between November 2013 and December 2014)^16^, 81.1% in Vietnam (from July to September 2019)^21^, 47% in Uganda^22^, 57.3% in Indonesia (between March 2017 and February 2018)^23^, 72.5% in Brazil^24^, and 81% in China^25^. In Spain, colistin-resistant *E. coli* isolates from pig farms were 91.6% resistant to at least three different antimicrobial classes^8^; in addition, 100% of *mcr*-positive *E*. *coli* isolated from fecal samples of healthy pigs are MDR^9^.

In this study, colistin-resistance genes were detected in 28 isolates (75.7%). In these 28 *mcr*-positive *E. coli*, the *mcr-1* and *mcr-3* genes were detected whereas *mcr-2*, *mcr-4, mcr-5, mcr-6, mcr-7, mcr-8*, *mcr-9* and *mcr-10* genes were not detected. Eight (21.6%) were *mcr-1* carriers, 10 (27.0%) were *mcr-3* carriers and 10 (27.0%) demonstrated co-occurrence of *mcr-1* and *mcr-3* isolates. The *mcr-1* is the first gene reported and is globally distributed whereas others are less distributed^8^. In recent studies, the *mcr-3* was also found along with *mcr-1* 0.43% and 3% respectively in *E. coli* isolates from pig farms in Thailand and Vietnam^9, 26^. This is the first result that showed the *mcr-3* gene found with a high rate (54%), which should be an alert for the rapid emergence of *mcr-3*-mediated colistin resistance in pig farm in Thailand.

On the other hand, one of the 28 colistin-resistant isolates did not carry the *mcr* gene (tested *mcr* genes) in this study. On the contrary, one *mcr*-positive (*mcr-1* and *mcr-3*) isolate was susceptible to colistin, which is in agreement with an earlier report by García et al.^8^ in which six of 143 colistin-resistant isolates did not contain *mcr* genes and three *mcr*-positive isolates were susceptible to colistin. This inactive form of *mcr* might be explained by the occurrence of an insertion of a 1.7 Kb IS1294b element into *mcr-1*^8^. It does not escape our attention that there was one *mcr*-negative colistin-resistant isolate. There might possibly be an alternative colistin-resistant mechanism that does not involve the *mcr* gene. Such mechanism would be of interest for further study. Nonetheless, the correlation between genotypes and phenotypes of colistin resistance was considered strongly statistically significant (κ = 0.853; *p* < 0.001). The high proportion of *mcr*-positive isolates (75.7%) is a risk for public health. In addition, 75.7% of isolates carried the *int1* gene and 24/28 (85.7%) *int1*-positive isolates carried the *mcr* gene. It has been reported that the presence of the *int1* gene (a mobile genetic element) in pathogens, may increase the ability to transfer resistance genes to other bacteria in the environment^27^. ERIC-PCR profiles of isolated *E. coli* showed that isolates have diverse genetic structures. A total of 37 isolates were differentiated into thirteen groups with 65% similarity. Beside various gene sources, the rapid and easy genetic modification to adapt in *E. coli* may also cause genetic diversity. Frequent use of antibiotics in pig farms creates selective pressure leading to genetic modification in *E. coli*. In addition, each pig farm may have its own treatment approach for the selection of antibiotics in order to control piglet diarrhea. This can also result in the genetic diversity of isolates among pig farms^28^. In addition*,* Guenther et al. showed that antibiotic-resistant strains can transmit resistance genes to other pathogenic bacteria, especially to human pathogens^29^. It is worth noting that colistin is an antibiotic that is widely used in both humans and animals. Thus, the possibility of spreading the *mcr* gene from animals to humans is a serious public health concern. Bacteria carrying *mcr* gene have been found in humans and animals in many countries. Recently, it has been demonstrated that *E. coli* strains carrying *mcr-1* and *mcr-3* genes were found not only in pig feces, but also from contaminated pig carcasses and pork^30, 31^. This indicates a high risk of spreading bacteria-harboring *mcr* genes to humans and other environments.

## Conclusion

In the present study, multidrug-resistance (MDR) *E. coli* was found in a high proportion of fecal sample (97.3%) isolated from diarrheic piglets from pig farms in Thailand. From 10 *mcr* (*1–10*) genes tested, a large number of isolates harbored colistin-resistance genes *mcr-1* and *mcr-3*. The correlation between the colistin resistance phenotype and genotype among isolates was significant (*p* < 0.001). Taken together, the results of this study provide informative scientific evidence regarding bacterial resistance to antibiotics in pig farms, and it should also raise public health awareness regarding transmitting resistance gene from animals to humans.

## Supplementary Information

Supplementary Table S1.

Supplementary Figure S1.

## Data Availability

The datasets generated and/or analyzed during the current study are available from the corresponding author on reasonable request.

## Abbreviations

AMC: Amoxicillin: clavulanic acid
AMX: Amoxicillin
ATCC: American type culture collection
CAZ: Ceftazidime
CEF: Ceftiofur
CLSI: Clinical and Laboratory Standards Institute
CN: Gentamicin
CRO: Ceftriaxone
CT: Colistin
ENR: Enrofloxacin
DAEC: Diffusely adherent *E. coli*
EAEC: Enteroaggregative *E. coli*
EIEC: Enteroinvasive *E. coli*
EPEC: Enteropathogenic *E. coli*
ERIC-PCR: Enterobacterial repetitive intergenic consensus polymerase chain reaction
ETEC: Enterotoxigenic *E. coli*
MCRPE: *mcr*-Positive *Escherichia coli*
MDR: Multidrug resistance
MHB: Mueller Hinton broth
MIC: Minimal inhibitory concentration
OTC: Oxytetracycline
PCR: Polymerase chain reaction
STEC: Shiga toxin-producing *E. coli*
SXT: Trimethoprim/sulfamethoxazole
TBE: Tris–borate-EDTA
UPGMA: Unweighted pair-group method using arithmetic averages
