# Supplementary material for: Antibiotic resistant Escherichia coli from diarrheic piglets from pig farms in Thailand that harbor colistin-resistant mcr genes
Source: Sci Rep. 2022 May 31;12:9083. doi: 10.1038/s41598-022-13192-3 (PMC9156692; doi:10.1038/s41598-022-13192-3)
Supplement: Supplementary file 1 — Supplementary Table S1. [file 41598_2022_13192_MOESM1_ESM.docx]

**Antibiotic Resistant *Escherichia coli* from Diarrheic Piglets from Pig Farms in Thailand that Harbor Colistin-resistant *mcr* Genes**

Luong Thi Yen Nguyet^1^, Krittika Keeratikunakorn^1^, Kampon Kaeoket^1*^, Natharin Ngamwongsatit^1,2*^

^1^Department of Clinical Sciences and Public Health, Faculty of Veterinary Science, Mahidol University, Nakhon Pathom, Thailand

^2^Laboratory of Bacteria, Veterinary Diagnostic Center, Faculty of Veterinary Science, Mahidol University, Nakhon Pathom, Thailand

*Corresponding authors: Kampon Kaeoket, Natharin Ngamwongsatit

**Table S1.** Primers used in this study.

| **Target** | **Primer name** | **Sequence (5’-3’)** | **Product size (bp)** | **Source** |
| --- | --- | --- | --- | --- |
| **Virulence genes identification** | | | | |
| Set 1A | | | | |
| *ipaH* | ipaHF | 5’-CTG GCT GAT GCC GTG ACA G-3’ | 801 | This study |
|  | ipaHR | 5’-GCT GTT CAG TCT CAC GCA TC-3’ |  |  |
| *eaeA* | eaeAF | 5’-GCG ATT ACG CGA AAG ATA CC-3’ | 677 | This study |
|  | eaeAR | 5’-GAT AAC GGA ACT GCA TTG AGT-3’ |  |  |
| *lt* | LT-F | 5’-ATG ACG GAT ATG TTT CCA CTT CTC-3’ | 393 | [32] |
|  | LT-R | 5’-AAC CTT GTG GTG CAT GAT GAA TCC-3’ |  |  |
| *bfpA* | bfpAF | 5’-AGT CGC AGA ATG CTA TTT CAG AAG-3’ | 322 | This study |
|  | bfpAR | 5’-TTT TCG CCA GAG ATA TTA ACA CCG-3’ |  |  |
| *sth* | STh-F | 5’-TTC ACC TTT CGC TCA GGA TGC TA-3’ | 168 | [32] |
|  | STh-R2 | 5’-CAC CCG GTA CAA GCA GGA TT-3’ |  |  |
| Set 1B |  |  |  |  |
| F41 | F41F | 5’-GGA GCG GGT CAT ATT GGT AA-3’ | 941 | [33] |
|  | F41R | 5’-CTG CAG AAA CAC CAG ATC CA-3’ |  |  |
| F4 | F4F | 5’-GCC TGG ATG ACT GGT GAT TT-3’ | 715 | [33] |
|  | F4R | 5’-TCT GAC CGT TTG CAA TAC CC-3’ |  |  |
| F6 | F6F | 5’-GCG TGC ATC GAA ATG AGT T-3’ | 589 | [33] |
|  | F6R | 5’-GGT GGT TCC GAT GTA TGC TT-3’ |  |  |
| F18 | F18F | 5’-CTT TCA CAT TGC GTG TGG AG-3’ | 441 | [33] |
|  | F18R | 5’-ATT CGA CGC CTT AAC CTC CT-3’ |  |  |
| F5 | F5F | 5’-TTG GGC AGG CTG CTA TTA GT-3’ | 222 | [33] |
|  | F5R | 5’-TAG CAC CAC CAG ACC CAT TT-3’ |  |  |
| Set 2A |  |  |  |  |
| *stx1A* | Stx1AF | 5’-TCT GCA ATA GGT ACT CCA TTA CAG-3’ | 724 | This study |
|  | Stx1AR | 5’-CCG GAC ACA TAG AAG GAA AC-3’ |  |  |
| *stx2A* | Stx2AF | 5’-TTG ACC ATC TTC GTC TGA TTA TTG-3’ | 541 | This study |
|  | Stx2AR | 5’-CTG ATG ATG GCA ATT CAG TAT AAC-3’ |  |  |
| *pCVD432* | pCVDF | 5’-CTC TGG CGA AAG ACT GTA TC-3’ | 463 | This study |
|  | pCVDR | 5’-CAT CTC TAC ATC AAG AGC AG-3’ |  |  |
| *aggR* | aggRF | 5’-GTA TAC ACA AAA GAA GGA AGC-3’ | 254 | [34] |
|  | aggRR | 5’-ACA GAA TCG TCA GCA TCA GC-3’ |  |  |
| *stp* | STp-F | 5’-TTA ATA ACA TCC AGC ACA GGC AGG-3’ | 176 | [32] |
|  | STp-R | 5’-TCC CCT CTT TTA GTC AGT CAA CTG-3’ |  |  |
| Set2B |  |  |  |  |
| *stx2e* | Stx2eF | 5’- TGG TGT CAG AGT GGG GAG AA-3’ | 351 | [33] |
|  | Stx2eR | 5’- TAC CTT TAG CAC AAT CCG CC-3’ |  |  |
| *astA* | astAF | 5’- CCA TCA ACA CAG TAT ATC CGA-3’ | 111 | [34] |
|  | astAR | 5’- GGT CGC GAG TGA CGG CTT TGT-3’ |  |  |
| Set 3B |  |  |  |  |
| *aidA* | aidAF | 5’- TGG TGG GAA AAC CAC TGC TA-3’ | 771 | [33] |
|  | aidAR | 5’- TAG CCG CCA TCA CTA ACC AG-3’ |  |  |
| *paa* | paaF | 5’- CCA TAA AGA CAG CTT CAG TGA AAA-3’ | 162 | [35] |
|  | paaR | 5’- GTA TTA CTG GTA CCA CCA CCA TCA-3’ |  |  |
| **Drug resistance genes** | | | | |
| *mcr-1* | MCR1F | 5’-AGT CCG TTT GTT CTT GTG GC-3’ | 320 | [36] |
|  | MCR1R | 5’-AGA TCC TTG GTC TCG GCT TG-3’ |  |  |
| *mcr-2* | MCR2F | 5’-CAA GTG TGT TGG TCG CAG TT-3’ | 715 | [36] |
|  | MCR2R | 5’-TCT AGC CCG ACA AGC ATA CC-3’ |  |  |
| *mcr-3* | MCR3F | 5’-AAA TAA AAA TTG TTC CGC TTA TG-3’ | 929 | [36] |
|  | MCR3R | 5’-AAT GGA GAT CCC CGT TTT T-3’ |  |  |
| *mcr-4* | MCR4F | 5’-TCA CTT TCA TCA CTG CGT TG-3’ | 1116 | [36] |
|  | MCR4R | 5’-TTG GTC CAT GAC TAC CAA TG-3’ |  |  |
| *mcr-5* | MCR5F | 5’-ATG CGG TTG TCT GCA TTT ATC-3’ | 1644 | [36] |
|  | MCR5R | 5’-TAC TTG TGG TTG TCC TTT TCT G-3’ |  |  |
| *mcr-6* | MCR6F | 5’-GTC CGG TCA ATC CCT ATC TGT-3’ | 556 | [37] |
|  | MCR6R | 5’-ATC CGG GAT TGA CAA GTA C-3’ |  |  |
| *mcr-7* | MCR7F | 5’-TGC TAC AGC CCT TTT CGT-3’ | 892 | [37] |
|  | MCR7R | 5’-TTC ATC TGC GCC ACC TCG T-3’ |  |  |
| *mcr-8* | MCR8F | 5’-AAC CGC CAG AGC ACA GAA TT-3’ | 667 | [37] |
|  | MCR8R | 5’-TTC CCC CAG CGA TTC TCC AT-3’ |  |  |
| *mcr-9* | MCR9F | 5´-AGA ACA TGC ACG GAA CGG AT-3´ | 183 | [38] |
|  | MCR9R | 5´-CTC ACG AAA AAC CCA CGC TG-3´ |  |  |
| *mcr-10* | MCR10F | 5’-AGC CGT CTT GAA CAT GTG AG-3’ | 744 | [39] |
|  | MCR10R | 5’-CAT ACA GGG CAC CGA GAC TG-3’ |  |  |
| *int1* | INT1-F | 5’-CTC CCG CAC GAT GAT CGT-3’ | 450 | This study |
|  | INT1-R | 5’-TTG CGT GAG CGC ATA CGC-3’ |  |  |

**References for supplementary table**

32. Puiprom, O. *et al.* Identification of colonization factors of enterotoxigenic *Escherichia coli* with PCR-based technique. *Epidemiol. Infect.* **138**, 519-524 (2010).

33. Lee, S. I., Kang, S. G., Kang, M. L. & Yoo, H. S. Development of multiplex polymerase chain reaction assays for detecting enterotoxigenic *Escherichia coli* and their application to field isolates from piglets with diarrhea. *J. Vet. Diagn. Invest.* **20**, 492-496 (2008).

34. Aslani, M. M., Alikhani, M. Y., Zavari, A., Yousefi, R. & Zamani, A. R. Characterization of enteroaggregative *Escherichia coli* (EAEC) clinical isolates and their antibiotic resistance pattern. *Int. J. Infect. Dis.* **15**, 136-9 (2011).

35. Zhang, W., Zhao, M., Ruesch, L., Omot, A. & Francis, D. Prevalence of virulence genes in *Escherichia coli* strains recently isolated from young pigs with diarrhea in the US. *Vet. Microbiol.* **123**, 145-152 (2007).

36. Rebelo, A. R. *et al.* Multiplex PCR for detection of plasmid-mediated colistin resistance determinants, *mcr-1*, *mcr-2*, *mcr-3*, *mcr-4* and *mcr-5* for surveillance purposes. *Euro Surveill.* **23**, 1-11 (2007).

37. Wetzker, W. *et al.* Extended-spectrum beta-lactamase (ESBL)-producing *Escherichia coli* isolated from flies in the urban center of Berlin, Germany. *Int. J. Environ. Res. Public Health* **16**, 16-9 (2019).

38. Khanawapee, A. *et al.* Distribution and molecular characterization of *Escherichia coli* harboring *mcr* genes isolated from slaughtered pigs in Thailand. *Microb. Drug. Resist.* **27**, 971-9 (2021).

39. Xu, T. *et al.* Identification of *mcr-10* carried by self-transmissible plasmids and chromosome in *Enterobacter roggenkampii* strains isolated from hospital sewage water. *Environ. Pollut.* **268**, 115706 (2021).
