# Supplementary material for: Antibiotic resistant Escherichia coli from diarrheic piglets from pig farms in Thailand that harbor colistin-resistant mcr genes
Source: Sci Rep. 2022 May 31;12:9083. doi: 10.1038/s41598-022-13192-3 (PMC9156692; doi:10.1038/s41598-022-13192-3)

**Antibiotic Resistant *Escherichia coli* from Diarrheic Piglets from Pig Farms in Thailand that Harbor Colistin-resistant *mcr* Genes**

Luong Thi Yen Nguyet^1^, Krittika Keeratikunakorn^1^, Kampon Kaeoket^1*^, Natharin Ngamwongsatit^1,2*^

^1^Department of Clinical Sciences and Public Health, Faculty of Veterinary Science, Mahidol University, Nakhon Pathom, Thailand

^2^Laboratory of Bacteria, Veterinary Diagnostic Center, Faculty of Veterinary Science, Mahidol University, Nakhon Pathom, Thailand

*Corresponding authors: Kampon Kaeoket, Natharin Ngamwongsatit

**Supplementary Figure 1.** The original ERIC-PCR gels of 37 *E. coli* isolates


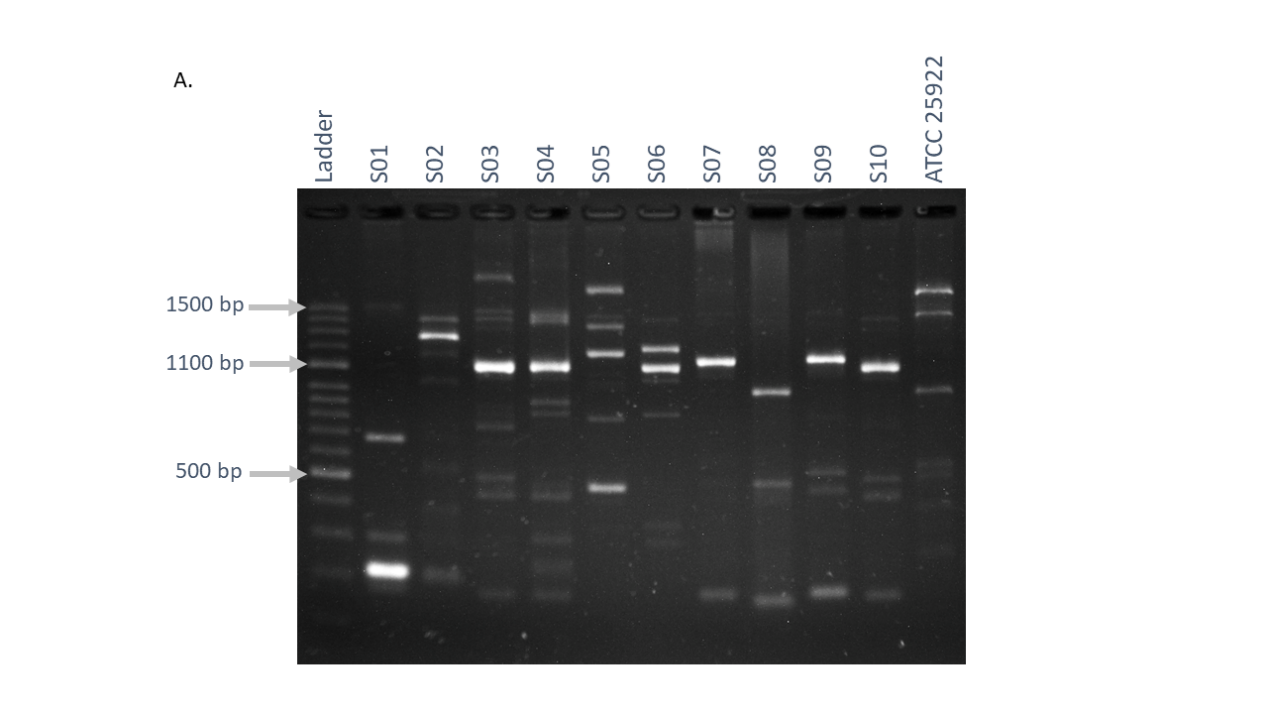


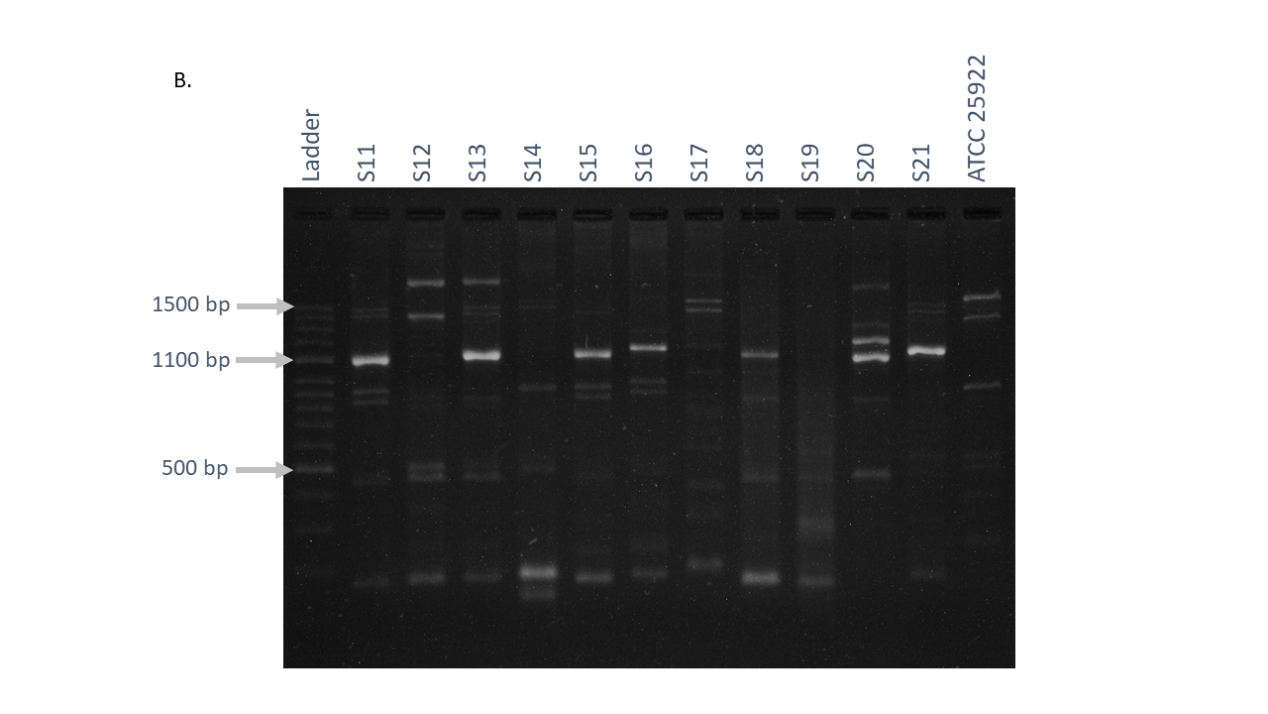


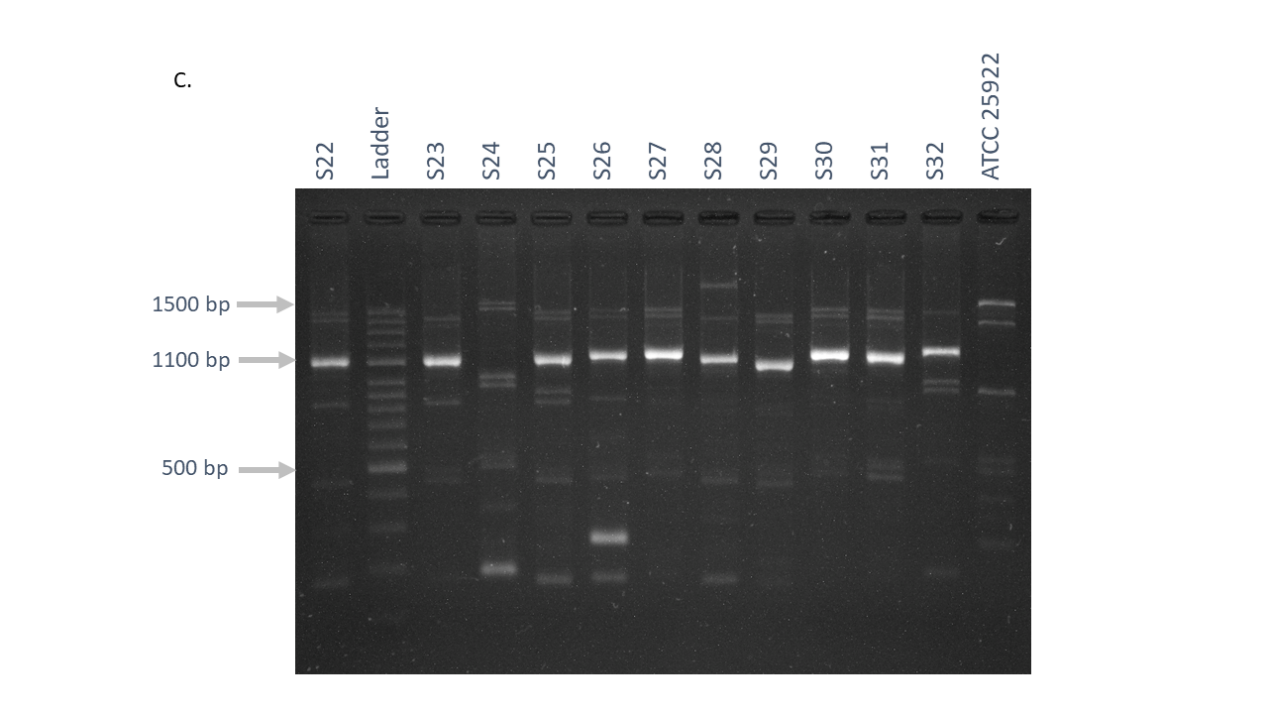


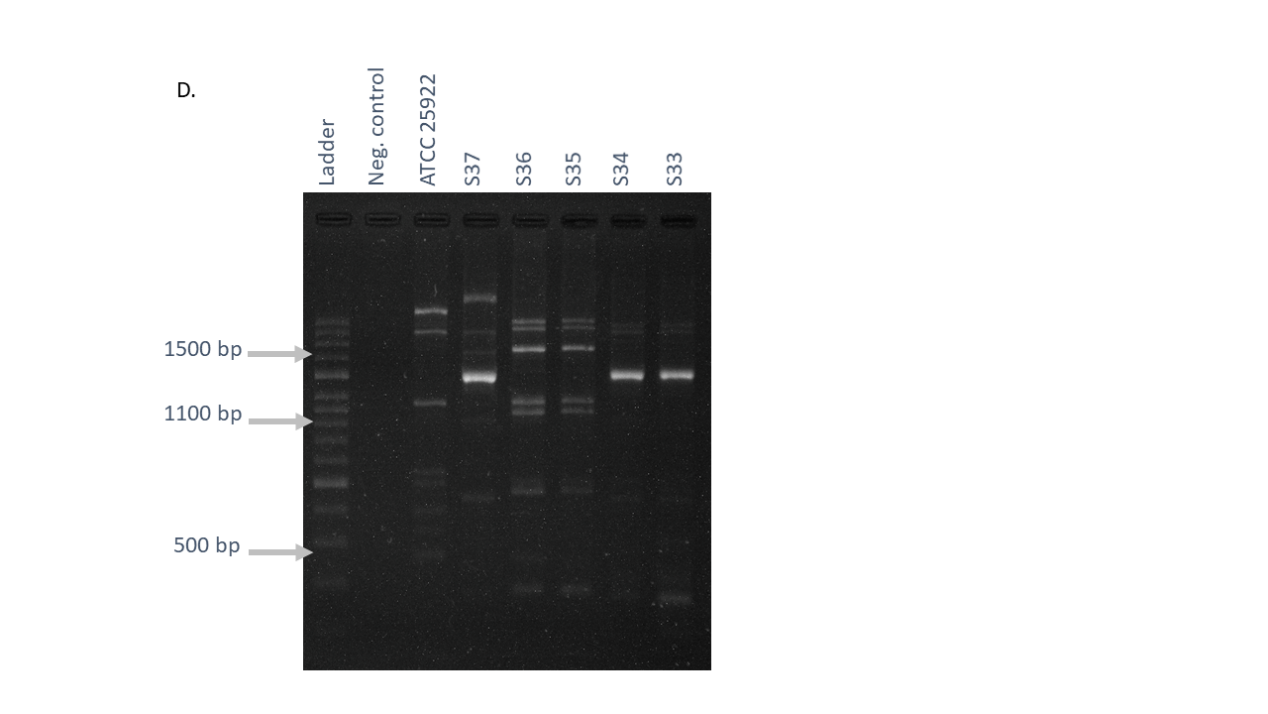

Supplement: Supplementary file 2 — Supplementary Figure S1. [file 41598_2022_13192_MOESM2_ESM.docx]
